# APOBEC3G levels predict rates of progression to AIDS

**DOI:** 10.1186/1742-4690-4-20

**Published:** 2007-03-20

**Authors:** Xia Jin, Hulin Wu, Harold Smith

**Affiliations:** 1 Departments of Medicine, University of Rochester, Rochester, New York 14642, USA; 2 Departments of Microbiology and Immunology, University of Rochester, Rochester, New York 14642, USA; 3 Department of Biostatistics and Computational Biology, University of Rochester, Rochester, New York 14642, USA; 4 Department of Biochemistry and Biophysics, University of Rochester, Rochester, New York 14642, USA

## Abstract

**Background:**

APOBEC3G (hA3G) is a newly discovered cellular factor of innate immunity that inhibits HIV replication *in vitro*. Whether hA3G conferrs protection against HIV *in vivo *is not known. To investigate the possible anti-HIV activity of hA3G *in vivo*, we examined hA3G mRNA abundance in primary human cells isolated from either HIV-infected or HIV-uninfected individuals, and found that hA3G mRNA levels follow a hierarchical order of long-term nonprogressors>HIV-uninfected>Progressors; and, hA3G mRNA abundance is correlated with surrogates of HIV disease progression: viral load and CD4 count. Another group later confirmed that HIV-infected subjects have lower hA3G mRNA levels than HIV-uninfected controls, but did not find correlations between hA3G mRNA levels and viral load or CD4 count. These conflicing results indicate that a more comprehensive, conclusive investigation of hA3G expression levels in various patient cohorts is urgently needed.

**Presentation of the hypothesis:**

For exploring whether hA3G abundance might influence HIV disease progression, we have formulated a hypothesis that inlcudes two parts: a) *in vivo*, the basal hA3G mRNA expression level per PBMC is a constant – with minor physiologic fluctuations – determined by host genetic and epigenetic elements in a healthy individual; and that the basal hA3G mRNA expression levels in a population follow a Normal (or Gaussian) distribution; b) that although HIV infects randomly, it results in more rapid disease progression in those with lower hA3G mRNA levels, and slower disease progression in those with higher hA3G mRNA levels.

**Testing the hypothesis:**

This hypothesis could be tested by a straighforward set of experiments to compare the distribution of hA3G mRNA levels in HIV-uninfected healthy individuals and that in HIV-infected, antiretroviral therapy-naïve subjects who are at early and late stages of infection.

**Implication of the hypothesis:**

Testing this hypothesis will have significant implications for biomedical research. a) It will link hA3G to the mechanisms underlying slower disease progression in long-term nonprogressors. And, b) It may help to establiseh a new prognostic marker, the hA3G abundance measurement, for HIV-infected patients.

## Background

In the absence of antiretroviral therapy, most HIV-infected individuals die of AIDS within 8–10 years of infection. Some of them, however, have a substantially slower rate of disease progression and have been categorized as long term nonprogressors (LTNPs), who are usually clinically asymptomatic, and having high CD4 counts and low HIV viremia levels [1]. These LTNPs offered unique opportunities to study correlates of protective immunity. Potential protective mechanisms in LTNPs include infection by defective or less fit HIV variants, having strong host immune responses, and possession of unique host genetic elements including CCR5 genotype and HLA haplotypes [1-19]. Current consensus is that each known factor only plays some degree of protection, and unknown protective host factors may yet to be discovered.

APOBEC3G (apolipoprotein B mRNA-editing enzyme, catalytic polypeptide-like 3G; also known as CEM15, or hA3G) is a novel cellular factor of innate immunity that inhibits HIV replication *in vitro *by causing G to A hypermutations, and consequently reduced relative infectivity of each virus produced by infected cells [20-22]. HIV counters hA3G activity by using Vif protein to bind and target hA3G protein for enhanced degradation through proteasomal pathways [21,22]. hA3G also has antiviral activities against other viruses including SIV, hepatitis B virus (HBV) and murine leukemia virus (MLV) [23-32]. Some recent studies, however, suggest that causing G to A mutations may not be the only mechanism by which hA3G exercises its antiviral activity, at least *in vitro *[25,33]. Whereas others reported that hA3G induced G to A hypermutations might be a major mechanism of virological control *in vivo *[34]. Additionally, it was reported that the activation of peripheral blood mononuclear cell (PBMC) will modified the hA3G from a low-molecular-mass (LMM) complex to a high-molecular-mass (HMM) complex, thus abrogating its antiviral effect. It was also suggested that in addition to the known mechanism of making G to A hypermutations, the LMM hA3G might exert post-entry restriction of HIV replication in resting primary CD4+T cells and monocytes [35].

Overall, there is currently a relative paucity of human research data. We propose a hypothesis that reconciles these published data using human cells [34,36,37]. Testing this hypothesis will have implications for a better undersanding of the HIV pathogenesis; the development of a new diagnostic tool; as wells as providing scientific basis for the therapeutic strategy that targets Vif protein or Vif-hA3G interections.

## Presentation of the hypothesis

**Figure 1:**
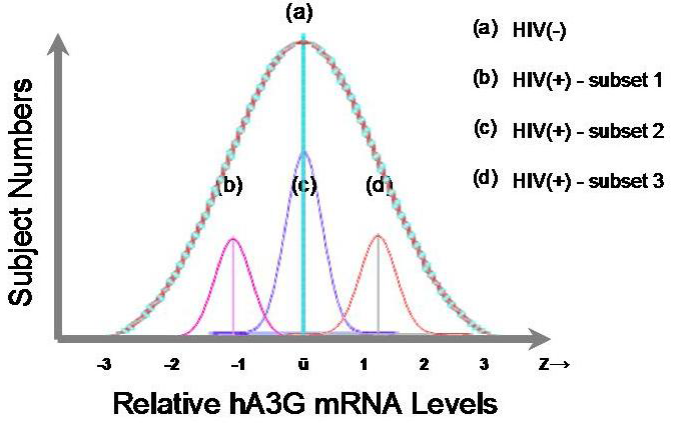
**Schematic illustration of the hypothesis**. We hypothesize that in human population hA3G mRNA levels (x-axis) follow a Normal distribution that has two major characteristics: 1) the hA3G mRNA levels of most subjects concentrate around the mean value of ū; and, 2) the remaining subjects have values falling on either side of the mean (as a value of "z"). Distribution "a" illustrates HIV-uninfected controls (HIV-). HIV-infected subjects could be stratified into low (HIV+ subset 1), medium (HIV+ subset 2), and high hA3G subsets based on relative hA3G mRNA abundance (HIV+ subset 3). Each group's hA3G levels still follow a Normal distribution (distribution b, c, and d, respectively).

**Figure 2:**
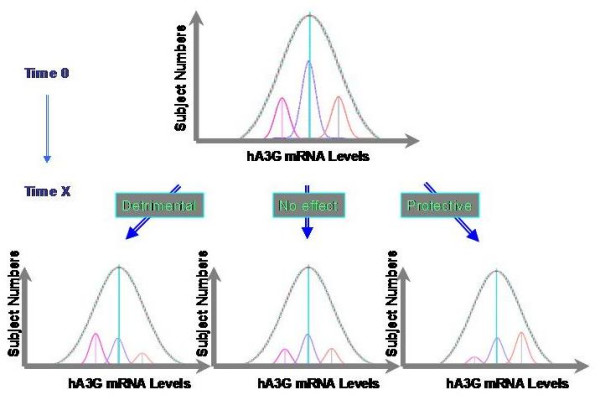
**Testable predictions of the hypothesis**. After a period of HIV infection (at Time X), there will be three outcomes for the distribution. If hA3G level has no effect on HIV disease progression, the distributions of hA3G level in HIV-infected and HIV-uninfected individuals should overlap (No effect, middle). If hA3G plays a protective role, its distribution in HIV-infected patients will shift to the right in comparison to that of HIV-uninfected controls (Protective, right). If hA3G plays a detrimental role, then its distribution in HIV-infected patients will shift to the left in comparison to HIV-uninfected individuals (Detrimental, left).

Why is there individual variation in hA3G mRNA expression level in recent reports [36,37]? We hypothesize that *in vivo*, the basal hA3G mRNA expression level per PBMC is a constant – with minor physiologic fluctuations – determined by host genetic and epigenetic elements in a healthy individual; and that the basal hA3G mRNA expression levels in a population follow a Normal (or Gaussian) distribution. Figure 1 uses distribution "a" to illustrate two major features of the hypothesis: 1) the hA3G mRNA levels of most subjects concentrate around the mean value of ū; and, 2) the remaining subjects have values falling on either side of the mean (as a value of "z"). The hypothesis would predict the following scenario:

At the initial time of infection (Time 0, Fig 2), HIV randomly infects individuals with differential hA3G mRNA abundance. According to the relative hA3G mRNA abundance, these HIV-infected subjects could be stratified into low, medium, and high hA3G subsets. Each patient group's hA3G levels will still form a Normal distribution (distribution b, c, and d, respectively; Fig 1). Collectively, though, the distribution of hA3G mRNA abundance among HIV-infected and uninfected subjects will overlap initially, albeit the population size of HIV-infected will be smaller than that of the HIV-uninfected. After a period of HIV infection (Time X, Fig 2), when patients' viremia reach steady-state levels and their clinical stages become stable, there will be several testable outcomes for the distribution. If hA3G level has no effect on HIV disease progression, the distributions of hA3G level in HIV-infected and HIV-uninfected individuals will overlap because subsets of HIV-infected patients who have varying hA3G levels should have similar attrition rates. If hA3G plays a protective role, one should expect increased survival of those with higher hA3G levels, such that the overall hA3G levels will shift to the right in comparison to that of normal HIV-uninfected controls. If hA3G plays a detrimental role, then its distribution in HIV-infected patients will shift to the left (Fig. 2).

To generalize the hypothesis, we can designate a mean value for hA3G abundance to each subject population of interest. Let (i) the HIV-uninfected subjects maintained the same distribution (as shown in Fig 1), with a mean of ū; (ii) individuals with higher hA3G mRNA abundance that are enriched for LTNPs have a mean of ā; and, (iii) other HIV-infected subjects have a mean of ē. Assuming the proportion of LTNP in the entire HIV-infected population is *W*, then we would have

ū = Wā + (1-W)ē.

This generalized model could be tested in studies described below in the "Testing the hypothesis" section. Our published results are in good agreement with these predictions. Specifically, in a cross-sectional study involving subjects who have been chronically HIV-infected for many years (14 ± 4 years for LTNPs, and 8 ± 6 years for the others), we have observed that LTNPs had much higher hA3G mRNA abundance than HIV-uninfected controls (ā > ū, and ā > ē), who had higher hA3G mRNA abundance than other HIV-infected progressors (ū > ē) [36]. If the number of LTNPs are very small (when *W *approaches 0), the mean of hA3G mRNA levels in HIV-infected progressors will be very close to that of HIV-uninfected subjects (ū = ē).

Our central hypothesis has one assumption that the basal hA3G mRNA expression levels in healthy control individual follow a Normal distribution. This could be easily tested using the Kolmogorov-Smirnov test (K-S test), which is one of the most frequently used nonparametric tests for examining whether an underlying probability distribution differs from a hypothesized distribution derived from a relative small sample size. The K-S statistics quantifies the discrepancy (D) between the experimental data and an ideal Normal distribution using the following equations. The empirical distribution function *F*_*n *_for *n *observations *y*_*i *_is defined as

Fn(x)=1n∑i=1n{1if yi≤x,0otherwise.
 MathType@MTEF@5@5@+=feaafiart1ev1aaatCvAUfKttLearuWrP9MDH5MBPbIqV92AaeXatLxBI9gBaebbnrfifHhDYfgasaacH8akY=wiFfYdH8Gipec8Eeeu0xXdbba9frFj0=OqFfea0dXdd9vqai=hGuQ8kuc9pgc9s8qqaq=dirpe0xb9q8qiLsFr0=vr0=vr0dc8meaabaqaciaacaGaaeqabaqabeGadaaakeaacqWGgbGrdaWgaaWcbaGaemOBa4gabeaakiabcIcaOiabdIha4jabcMcaPiabg2da9maalaaabaGaeGymaedabaGaemOBa4gaamaaqahabaWaaiqabeaafaqaaeGacaaabaGaeGymaedabaacbaGae8xAaKMae8NzayMaeeiiaaIaemyEaK3aaSbaaSqaaiabdMgaPbqabaGccqGHKjYOcqWG4baEcqGGSaalaeaacqaIWaamaeaacqWFVbWBcqWF0baDcqWFObaAcqWFLbqzcqWFYbGCcqWF3bWDcqWFPbqAcqWFZbWCcqWFLbqzcqGGUaGlaaaacaGL7baaaSqaaiabdMgaPjabg2da9iabigdaXaqaaiabd6gaUbqdcqGHris5aaaa@57C9@

The two one-sided K-S test statistics are given by

Dn+=max⁡(Fn(x)−F(x))Dn−=max⁡(F(x)−Fn(x))
 MathType@MTEF@5@5@+=feaafiart1ev1aaatCvAUfKttLearuWrP9MDH5MBPbIqV92AaeXatLxBI9gBaebbnrfifHhDYfgasaacH8akY=wiFfYdH8Gipec8Eeeu0xXdbba9frFj0=OqFfea0dXdd9vqai=hGuQ8kuc9pgc9s8qqaq=dirpe0xb9q8qiLsFr0=vr0=vr0dc8meaabaqaciaacaGaaeqabaqabeGadaaakqaabeqaaiabdseaenaaDaaaleaacqWGUbGBaeaacqGHRaWkaaGccqGH9aqpcyGGTbqBcqGGHbqycqGG4baEcqGGOaakcqWGgbGrdaWgaaWcbaGaemOBa4gabeaakiabcIcaOiabdIha4jabcMcaPiabgkHiTiabdAeagjabcIcaOiabdIha4jabcMcaPiabcMcaPaqaaiabdseaenaaDaaaleaacqWGUbGBaeaacqGHsislaaGccqGH9aqpcyGGTbqBcqGGHbqycqGG4baEcqGGOaakcqWGgbGrcqGGOaakcqWG4baEcqGGPaqkcqGHsislcqWGgbGrdaWgaaWcbaGaemOBa4gabeaakiabcIcaOiabdIha4jabcMcaPiabcMcaPaaaaa@57A8@

where *F*(*x*) is the hypothesized distribution, or another empirical distribution. The probability distributions of these two statistics do not depend on what the hypothesized distribution is, as long as it is continuous.

It should be emphasized that because hA3G's antiviral effect is part of the innate immunity, we believe that hA3G mRNA abundance per cell is mostly genetically determined, and it does not serve as a metric for CD4+ T cell count. Therefore while the hA3G mRNA levels may serve as a predictor of the rate of disease progression, it will not be a mere surrogate of CD4+ T cell count.

## Testing the hypothesis

To test whether hA3G mRNA expression levels in PBMCs follow a Normal distribution in a population, it is critical to perform its measurement in a sufficient number of subjects. We used the one-sample K-S test [38] to determine the required sample size. In the K-S test statistics, *D *is defined as the maximum absolute difference between the empirical distribution function and the estimated cumulative distribution function. From our preliminary study [36], we calculated that the *D *values for the HIV-negative (N), HIV-positive progressors (P), and LTNPs are 0.23, 0.28 and 0.28, respectively. To ensure that the population of HIV-infected subjects is big enough so that it will contain subjects with hA3G mRNA abundance similar to that of the HIV-infected P and LTNP, we conservatively set the maximum absolute differences to be 0.22, 0.26 and 0.26 for the N, P and LTNP subsets, respectively. If the type I error α = 0.05 and a minimum power of 80%, the calculated sample sizes are 66, 41 and 41 based on the method proposed by Massey [39]. Table 1 summarizes the required sample sizes for other possible values of *D *and power. Conservatively, we propose a sample size of 80 subjects for each testing group, so that we can detect a small difference of *D *= 0.2.

**Table 1:** Sample size requirement for testing a normal distribution

	α = 0.05, power				
	
*D*	0.05, 0.9	0.05, 0.8	0.05, 0.7	0.05, 0.6	0.05, 0.5
0.2	101	80	66	56	46
0.22	83	66	54	46	38
0.24	70	56	46	39	32
0.26	60	47	39	33	27
0.28	52	41	33	28	24

The experimental plan is relatively straightforward. Based on sample size calculation, we will study the hA3G mRNA levels in a total of 80 HIV-uninfected subjects, and 80 early HIV-infected subjects. We chose early HIV infected individuals because they are chronologically closest to the HIV-uninfected population. Once hA3G mRNA levels from all subjects are determined, their overall distribution will be analyzed using one-sample K-S test [38]. The hA3G mRNA distributions of HIV-uninfected and HIV-infected subjects will then be compared.

As a complementary approach, we will next select 80 chronically HIV-infected subjects who had been infected for more than five year without antiretroviral intervention, anticipating that this patient population had undergone selection over time by virological and host genetic factors. We expect that the population mean of hA3G levels in these HIV-infected patients shifted away from the mean value of HIV-uninfected controls. Dependent on whether hA3G plays a protective or a detrimental role *in vivo*, such a shift could be either to the right, or to the left.

To directly test whether HIV infection influences hA3G mRNA level, patients at different stages of infection will be enrolled into a highly-active antiretroviral therapy (HAART) treatment trial for 12 months. PBMC will be isolated from sequential blood samples (months 0, 3, 6 and 12) for measuring hA3G mRNA levels. It is expected that a majority of patients will be responsive to the HAART treatment that could significantly reduce the HIV viral load. If hA3G mRNA levels do not change significantly during the HAART treatment, then HIV-infection per se is unlikely to have direct impact on the hA3G expression; if they do change significantly, then the mechanisms responsible for the changes will need to be further studied.

One anticipated problem is that hA3G mRNA abundance might not follow a Normal distribution. Instead, it follows either a truncated Normal distribution, or other unimodal distributions. If so, we will test an alternative hypothesis that the distributions of hA3G mRNA abundance are the same in HIV-infected subjects as in HIV-uninfected controls. According to our sample size calculation, 80 subjects from each group will give 80% power for testing the alternative hypothesis at α = 0.05 and effect size of 0.1 (or 10%). This alternative hypothesis, if proven to be correct, can still explain our observations, and lends support to the idea that hA3G mRNA abundance is another good prognostic marker for HIV disease progression.

There are pros and cons of using different cell preparation for hA3G quantification. Not all human cells express hA3G. In PBMC used for our assay, however, several major subsets that constitute 60–70% of PBMC: CD4+ T cell, CD8+ T cell and monocytes all express hA3G. Ideally, one would want to perform the assay with purified CD4+ T cells that are the major target cells for HIV infection. The problem is that all known methods for purifying cells would require several hours of manipulation: either by the negative selection method which does not give high purity; or the positive selection method which may activate cells (and thus modulate hA3G mRNA levels) through specific binding antibody used for purification. Therefore, using unperturbed PBMC for assessing hA3G mRNA level may be a good compromise, albeit not a perfect solution.

## Implications of the hypothesis

If the hypothesis were true, it will have at least two significant implications for HIV research: a) It will elucidate an unrecognized mechanism responsible for slower disease progression in long term nonprogressors; and, b) It may help to establish a new prognostic marker, the hA3G aboundance measurement, for HIV-infected patients.

It is clear that HIV disease progression is determined by multiple factors. Testing our hypothesis will provide proof or refutation of the idea that hA3G mRNA level *in vivo *influences HIV disease progression, but it will not discern whether hA3G mRNA level is an independent predictor, or it has to work in concert with other host factors. One of the new host factors is the tripartite motif protein (TRIM)5α, which was initially found to restrain HIV-1 infection in monkeys [40]. Some variants of TRIM5α conferred modest protection against HIV-1 disease progression in humans [41,42]. Nevertheless, a conclusive study to determine the influence of hA3G mRNA level on HIV disease progression is evidently needed.

## List of abbreviations used

APOBEC3G (hA3G): apolipoprotein B mRNA-editing enzyme, catalytic polypeptide 3G; also known as CEM15, or hA3G.

LTNP: long term nonprogressors.

## Competing interests

The authors XJ and HW have no financial competing interests. HS is the CSO of the Oxagen, Inc., a company that develops antiviral reagents based on Vif and hA3G.

## Authors' contributions

XJ and HS performed a pilot study which contributes to the genesis of the hypothesis proposed in the current paper. XJ conceived and drafted the initial manuscript. The mathematical/statistical aspects of the hypothesis were aided by HW. All authors contributed to revision of the draft manuscript, read and approved the final manuscript.
